# Comparative Analysis of γ-Cyclodextrin, Perilla Oil, and Their Inclusion Complexes on Liver Injury and Dyslipidemia Associated with Elevated Gastrointestinal 12-Hydroxylated Bile Acid Levels

**DOI:** 10.3390/molecules30020281

**Published:** 2025-01-13

**Authors:** Keisuke Yoshikiyo, Hidehisa Shimizu, Edward G. Nagato, Satoshi Ishizuka, Tatsuyuki Yamamoto

**Affiliations:** 1 Institute of Agricultural and Life Sciences, Academic Assembly, Shimane University, 1060 Nishikawatsu-Cho, Matsue 690-8504, Shimane, Japan; 2 Graduate School of Natural Science and Technology, Shimane University, 1060 Nishikawatsu-Cho, Matsue 690-8504, Shimane, Japan; 3 Faculty of Life and Environmental Sciences, Shimane University, 1060 Nishikawatsu-Cho, Matsue 690-8504, Shimane, Japan; 4 The United Graduate School of Agricultural Sciences, Tottori University, 4-101 Koyama-Minami, Tottori 680-8553, Tottori, Japan; 5 Interdisciplinary Center for Science Research, Shimane University, 1060 Nishikawatsu-Cho, Matsue 690-8504, Shimane, Japan; 6 Institute of Environmental Systems Science, Academic Assembly, Shimane University, 1060 Nishikawatsu-Cho, Matsue 690-8504, Shimane, Japan; 7 Institute of Nature and Environmental Technology, Kanazawa University, Kakuma-machi, Kanazawa 920-1192, Ishikawa, Japan; 8 Research Faculty of Agriculture, Hokkaido University, Kita 9, Nishi 9, Kita-ku, Sapporo 060-8589, Hokkaido, Japan; 9 Raman Project Center for Medical and Biological Applications, Shimane University, 1060 Nishikawatsu-cho, Matsue 690-8504, Shimane, Japan

**Keywords:** Perilla oil, γ-cyclodextrin, inclusion complex, 12-hydroxylated bile acids, cholic acid, α-linolenic acid, eicosapentaenoic acid, fatty acid, liver injury, dyslipidemia

## Abstract

Our previous study demonstrated that γ-cyclodextrin (γ-CD)–perilla oil inclusion complexes increase plasma α-linolenic acid and eicosapentaenoic acid levels in healthy rats without adverse effects. The present study examined the effects of perilla oil, γ-CD, and their inclusion complexes on rats fed cholic acid (CA) to mimic the elevated gastrointestinal 12-hydroxylated (12OH) bile acid levels in high-fat diet-fed rats. Rats fed CA (CA group) tended to have higher AST, ALT, plasma total cholesterol (T-CHO), and triglyceride (TG) levels compared to controls fed a standard diet without CA. Rats fed CA and perilla oil (CA+LP group) showed a tendency for lower AST and plasma TG levels than those in the CA group. Rats fed CA and γ-CD (CA+CD group) had significantly higher AST, ALT, plasma T-CHO, and TG levels than the controls, indicating severe liver injury and dyslipidemia. Rats fed CA and the γ-CD–perilla oil inclusion complex (CA+IC group) had significantly lower AST and ALT levels than the CA+CD rats, with a trend towards lower plasma T-CHO and TG levels. Plasma α-linolenic acid and eicosapentaenoic acid levels were significantly higher in the CA+LP and CA+IC groups than in the controls and CA+CD groups. However, the CA+IC group tended to have lower α-linolenic acid levels and significantly lower eicosapentaenoic acid levels than the CA+LP group. This suggests an accelerated conversion of α-linolenic acid to eicosapentaenoic acid in the CA+IC group, which may contribute to the attenuation of liver injury and dyslipidemia. These findings suggest that γ-CD may exacerbate liver injury and dyslipidemia caused by elevated gastrointestinal 12OH bile acid levels, whereas γ-CD–perilla oil inclusion complexes may ameliorate these effects by altering fatty acid metabolism. Furthermore, we recommend evaluating γ-CD safety in both healthy and pathological models and carefully selecting compounds co-ingested with γ-CD.

## 1. Introduction

Bile acids are sterol-based biomolecules that are synthesized in the liver and excreted into the bile. Their main function in the gastrointestinal tract is to emulsify fat, break it down into small particles, thereby helping absorb fatty acids and glycerides [1]. Primary bile acids, which are synthesized from cholesterol in the liver, are absorbed from the end of the ileum after undergoing conversion processes such as deconjugation and dehydrogenation in the small intestine. As a result, approximately 95% of the bile acids secreted into the gastrointestinal tract are reused (enterohepatic circulation) [1,2,3]. The residual primary bile acids, including cholic acid (CA), that reach the large intestine are transformed by the intestinal microbiota (predominantly *Clostridium XIVa* and *XI* group bacteria) into secondary bile acids, including deoxycholic acid (DCA) derived from CA, which exhibit cytotoxicity even at physiological concentrations [3,4]. Disturbances in bile acid homeostasis have been shown to be associated with various physiological and pathological processes owing to their diverse regulatory functions and potential toxicity [5]. For instance, bile acids are increasingly being recognized as significant regulators of glucose and lipid metabolism, and energy homeostasis [6,7]. Specifically, elevated DCA production is implicated in the development and progression of gallstones [8], colorectal cancer [9,10], Parkinson’s disease [11] and atherosclerosis associated with chronic kidney disease [12,13]. In recent years, studies of bile acid metabolic disorders have focused on the observation that the ratio of 12-hydroxylated (12OH) bile acids, consisting of bile acids such as CA and its derivatives, are higher than that of non-12OH bile acids, consisting of bile acids such as chenodeoxycholic acid and its derivatives. For instance, a human study showed that a reduction in serum levels of 12OH bile acids correlates with improvements in body mass index, insulin sensitivity, and fatty liver [14]. In rats, plasma and fecal levels of 12OH bile acids are selectively increased by the consumption of ethanol and a high-fat diet, respectively [15,16]. An analysis using a murine model demonstrated that the elevation in 12OH bile acid production is attributable to an increase in the expression of sterol 12-alpha-hydroxylase (CYP8B1), a crucial enzyme in CA synthesis, which is induced by the disruption of the insulin signaling pathway [17]. Based on these findings, rats fed a diet containing 0.5 g/kg CA, which mimics the increased release of 12OH bile acids into the gut due to a high-fat diet, exhibited an elevation of blood pressure [18], an increase in aspartate aminotransferase (AST) and alanine aminotransferase (ALT) (markers of liver injury), and dyslipidemia [19].

Cyclodextrin (CD) is a cyclic oligosaccharide composed of six, seven, or eight glucopyranose units (α-, β-, or γ-CD, respectively) that have the ability to include a variety of guest molecules in their molecular cavity to form inclusion complexes [20,21,22]. The formation of these CD inclusion complexes can alter the physicochemical properties of the included molecules, such as their aqueous solubility and stability against auto-oxidation [20,23]. After oral administration, CDs exhibit limited absorption in the gastrointestinal tract and are predominantly excreted unchanged in feces, resulting in generally low toxicity [20]. Notably, γ-CD has been granted a generally recognized as safe (GRAS) status by regulatory authorities for use in food and pharmaceutical applications [20,24,25].

An experiment using healthy rats revealed that when perilla oil was ingested as an inclusion complex with γ-CD, the proportion of α-linolenic acid in the plasma significantly increased, and the proportion of eicosapentaenoic acid, a metabolite of α-linolenic acid, in the plasma also showed a tendency to increase compared to when perilla oil was ingested alone [23]. In addition, when healthy rats were given γ-CD alone, alanine aminotransferase (ALT), which is an indicator of liver injury, tended to decrease, but when given the inclusion complex of γ-CD and perilla oil, ALT decreased significantly compared with the control rats [26]. Based on these findings, liver injury and dyslipidemia caused by increased levels of 12OH bile acids in the gastrointestinal tract are expected to be improved more effectively by inclusion complexes with γ-CD than by perilla oil alone. In addition, although the safety of γ-CD has been established in healthy rats [20,24,25], its effects have not been evaluated under conditions characterized by elevated levels of 12OH bile acids in the gastrointestinal tract, such as those induced by a high-fat diet. Therefore, the aim of the present study was to examine the effects of γ-CD, perilla oil, and γ-CD–perilla oil inclusion complexes on rats fed CA, which mimics the increase in 12OH bile acid levels in the gastrointestinal tract. In particular, this study focused on the analysis of AST and ALT (which are also indicators of liver injury), plasma total cholesterol (T-CHO), triglycerides (TG), and glucose (GLU), and the composition ratios of fatty acids in plasma.

## 2. Results

### 2.1. General Condition of Animals

This study was conducted using the five groups shown in Table 1: the control group was the American Institute of Nutrition (AIN)-93G (AIN-93G) diet (CTRL group); rats fed a CA diet in which part of the sucrose in AIN-93G was replaced by CA at a weight of 0.05% (CA group); rats fed a CA+LP diet in which part of the sucrose in AIN-93G was replaced by CA at a weight of 0.05%, and 12.6% of the soybean oil in AIN-93G was replaced with perilla oil (CA+LP group); rats fed a CA+CD diet in which part of the sucrose in AIN-93G was replaced by CA at a weight of 0.05%, and all the cellulose in AIN-93G was replaced by γ-CD (CA+CD group); rats fed a CA+IC diet in which part of the sucrose, all of the cellulose, and 12.6% of the soybean oil in AIN-93G was replaced by CA at a weight of 0.05% and the γ-CD–perilla oil inclusion complexes (CA+IC group).

**Figure 1:**
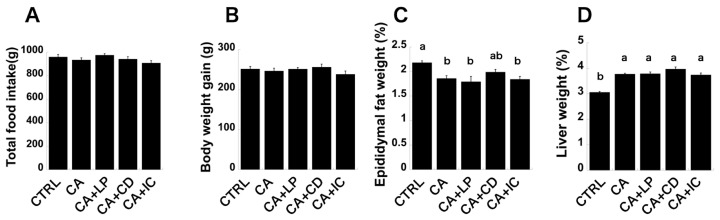
General observations of test animals. (**A**) Food consumption, (**B**) body weight gain, (**C**) relative epididymal fat weight to body weight, and (**D**) relative liver weight to body weight in the five feeding groups. Mean values are significantly different (*p* < 0.05) for groups with different letters.

The conditions of the animals in each group at the end of the experiment are summarized in Figure 1. The total amount of food consumed by each group during the 8-week experimental period was lowest in the CA+IC group and highest in the CA+LP group. Body weight gain was lowest in the CA+IC group and highest in the CA+CD group. Total food consumption and body weight gain tended to be lowest in the CA+IC group. However, there was no statistically significant difference in the total food consumption and body weight between the groups. The ratio of epididymal fat weight to body weight (%) was significantly lower in the CA, CA+LP, and CA+IC groups than in the CTRL group. In the CA+CD group, there was a tendency for the ratio of epididymal fat weight to body weight (%) to increase compared to the other groups that consumed diets containing CA, but there was no significant difference between this and the other groups. The ratio of liver weight to body weight (%) was the lowest in the CTRL group, which was significantly lower than in all groups containing CA in the diet. Among the groups consuming diets containing CA, the CA+IC group had the smallest mean value, while the CA+CD group had the highest mean value, but no statistically significant difference was observed between the groups. In addition to the fact that there was no significant difference in total food intake between the CTRL and CA groups, the increase in body weight and the ratio of liver weight to body weight due to the intake of the CA diet were consistent with the results of a previous study [19]. In addition, the addition of CA to the diet containing perilla oil, γ-CD, and γ-CD–perilla oil inclusion complexes did not elicit significant changes in total intake, body weight, or liver weight/body weight ratio. The absence of effects from these three components aligns with the findings of previous studies [23,26].

### 2.2. Plasma Parameters

**Figure 2:**
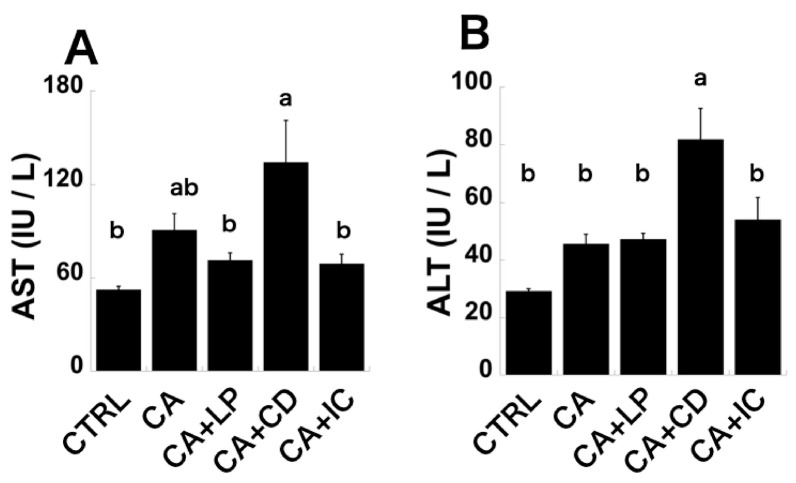
Levels of aspartate aminotransferase (AST) and alanine aminotransferase (ALT). (**A**) AST and (**B**) ALT of 5 feeding groups. Mean values are significantly different (*p* < 0.05) for groups with different letters.

**Figure 3:**
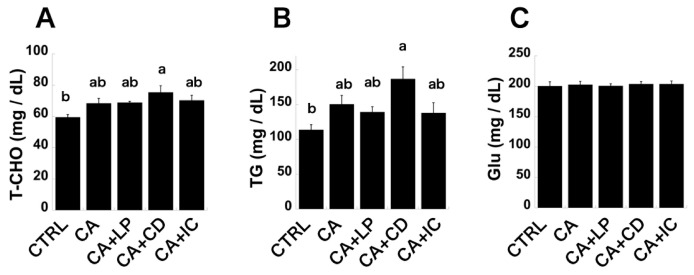
Plasma levels of total cholesterol (T-CHO), triglycerides (TG), and glucose (Glu). (**A**) T-CHO, (**B**) TG, and (**C**) Glu in five feeding groups. Mean values are significantly different (*p* < 0.05) for groups with different letters.

Statistical analysis of the five groups using the Tukey–Kramer post hoc analysis for the levels of AST and ALT, and plasma T-CHO and TG, did not show statistically significant differences between the CTRL and CA groups. However, when statistical analysis using the Student’s *t*-test was performed for these plasma parameters between the CTRL and CA groups to verify the reproducibility of the results of previous studies, a statistically significant difference was observed between the two groups, confirming reproducibility (Figure 2 and Figure 3) [19]. In addition, plasma glucose levels remained unchanged between the two groups, which is consistent with previous observations [19]. Figure 2A shows that the CTRL group exhibited the lowest AST levels, and an increasing trend was observed with the addition of CA to the diet. Although the CA+LP and CA+IC groups demonstrated marginally reduced AST levels compared with the CA group, this reduction was not statistically significant. The CA+CD group displayed a notable and statistically significant elevation, with AST levels exceeding twice that of the CTRL group. AST levels were significantly lower in the CA+IC group than in the CA+CD group. ALT levels exhibited an increasing trend in the CA group compared with in the CTRL group; however, no significant reduction in ALT levels was observed in the CA+LP and CA+IC groups. The elevation in ALT levels was statistically significant only in the CA+CD group, indicating an exacerbation of liver injury. This elevated level demonstrated significant improvement in the CA+IC group (Figure 2B). Plasma T-CHO and TG levels exhibited an increasing tendency in the CA group compared with the CTRL group; however, no improvement was observed in the plasma T-CHO levels of the CA+LP and CA+IC groups, and a decreasing trend was noted in the plasma TG levels. The CA+CD group showed a significant increase in these values compared with the CTRL group and a trend toward an increase compared with the CA group. The increase observed in the CA+CD group was significantly attenuated in the CA+IC group (Figure 3A,B). No difference was observed in the plasma GLU levels between the groups (Figure 3C). Taken together, perilla oil tended to improve AST and plasma TG levels, which showed an increasing trend in the CA group. In addition, in the CA+CD group, these parameters, which were further exacerbated, showed a statistically significant improvement when perilla oil was enclosed in γ-CD. Therefore, the adverse effects of γ-CD may be alleviated by the perilla oil included in γ-CD.

### 2.3. The Composition Ratios of Fatty Acids in Plasma

The plasma fatty acid composition ratios at the end of the feeding experiments are listed in Table 2. The composition ratios of fatty acids are expressed as the percentage of moles of a particular fatty acid out of the total number of moles of all fatty acids measured. In this experiment, the CTRL group, which consumed AIN-93G, served as the baseline for representing the plasma fatty acid composition of healthy rats. Of the nine fatty acids measured, no statistically significant differences were observed between the groups for palmitic acid, linoleic acid, and docosahexaenoic acid; however, statistically significant differences were observed between the groups for the other six fatty acids. The percentage composition of palmitoleic acid was lowest in the CTRL group and exhibited a more than two-fold increase in all groups containing CA in the diet. In addition, an upward trend was noted in the CA+CD group compared with the other groups. However, the percentage of stearic acid was significantly lower in all groups than in the CTRL group. Oleic acid levels were significantly higher in the CA, CA+CD, and CA+IC groups than in the CTRL group; however, no significant difference was observed between the CTRL and CA+LP groups. Furthermore, oleic acid levels were significantly lower in the CA+LP group than in the CA+IC group. The percentage of α-linolenic acid, the main component of perilla oil, was significantly higher in the CA+LP and CA+IC groups than in the other groups. However, the values for this parameter tended to decrease in the CA+IC group compared with those in the CA+LP group. The percentage of eicosapentaenoic acid, a metabolite of α-linolenic acid, was significantly elevated in the CA+LP and CA+IC groups compared with in the other groups, as was α-linolenic acid. However, the value of this parameter was significantly lower in the CA+IC group than in the CA+LP group. Arachidonic acid, a metabolite of linoleic acid, exhibited the highest ratio in the CTRL group and demonstrated a significant decrease in the CA+CD, CA+LP, and CA+IC groups compared with in the CTRL group. In addition, the CA+IC group exhibited a significant decrease in this parameter compared with the CA group and a downward trend compared with the CA+CD and CA+LP groups. Taken together, the ingestion of CA significantly increased the ratio of plasma palmitoleic acid to oleic acid and significantly decreased the ratio of plasma stearic acid compared with the CTRL group. Among the alterations in plasma fatty acid composition induced by the CA diet, the co-ingestion of CA and perilla oil significantly increased the ratio of plasma α-linolenic acid and eicosapentaenoic acid, while the ratio of plasma arachidonic acid and oleic acid exhibited a decreasing trend. The ratio of plasma palmitoleic acid and α-linolenic acid demonstrated an increasing tendency with the co-ingestion of CA and γ-CD. The co-ingestion of CA and γ-CD–perilla oil inclusion complexes significantly increased the ratio of plasma α-linolenic acid and eicosapentaenoic acid and significantly decreased arachidonic acid. A noteable observation among these results is that in comparing between the CA+LP and CA+IC groups that consumed diets with the same fatty acid composition, the ratio of oleic acid was significantly higher in the CA+IC group, whereas the ratios of α-linolenic acid and arachidonic acid exhibited a decreasing trend, and the ratio of eicosapentaenoic acid decreased significantly. This observation suggests that the presence of γ-CD in the diet induces a change in fatty acid metabolism in both groups.

## 3. Discussion

The present study demonstrated that AST and ALT and plasma T-CHO and TG levels were elevated in rats fed CA, which was consistent with a previous study in rats fed CA to mimic the elevated gastrointestinal 12OH bile acid levels observed in rats fed a high-fat diet [19]. Although the intake of perilla oil did not result in a significant improvement in liver injury and plasma T-CHO levels in rats that had consumed CA, a downward trend was observed in plasma TG levels. On the other hand, when γ-CD was consumed while the gastrointestinal 12OH bile acid levels were increased, liver injury and dyslipidemia worsened. Consumption of the γ-CD–perilla oil inclusion complex significantly improved liver injury, and dyslipidemia tended to improve under these conditions. Plasma α-linolenic acid and eicosapentaenoic acid levels increased significantly when subjects consumed perilla oil or the γ-CD–perilla oil inclusion complex; however, fatty acid metabolism differed between the two cases. This change in fatty acid metabolism may have had an effect on the improvement of liver injury and dyslipidemia, which were exacerbated when γ-CD was consumed when the gastrointestinal 12 OH bile acid levels increased. This study did not elucidate the mechanism by which γ-CD ingestion under conditions of elevated gastrointestinal 12OH bile acid levels exacerbate liver injury and dyslipidemia or the mechanism by which the γ-CD–perilla oil inclusion complex ameliorates these conditions. In addition, the influence of 12OH bile acids, including CA, on the γ-CD–perilla oil inclusion complex in the gastrointestinal tract has not yet been clarified. Therefore, addressing these unresolved questions is crucial to enhance our understanding of the findings of the present study.

In the CA+CD diet, all the cellulose in the CA diet was replaced with γ-CD. Consequently, by comparing the CA and CA+CD groups, it is possible to elucidate the effect of γ-CD intake when the levels of 12OH bile acids, such as CA, in the gastrointestinal tract are elevated. AST levels tended to increase in the CA group compared with those in the CTRL group and increased significantly in the CA+CD group. The CA+CD group also showed a tendency to increase compared with the CA group. Regarding ALT levels, an increasing trend was observed in the CA group compared with the CTRL group, and a significant increase was noted in the CA+CD group. In addition, a significant increase was observed in the CA+CD group compared with the CA group. These trends in AST and ALT levels suggest that the progression of liver injury caused by CA intake was exacerbated by the simultaneous intake of γ-CD. In the CA group, the levels of plasma TG and T-CHO tended to increase compared with those in the CTRL group, and this increase was more pronounced in the CA+CD group, with a statistically significant difference observed in comparison with the CTRL group. In the comparison between the CA and CA+CD groups, both parameters showed an increasing trend in the CA+CD group. In our previous study, we used a feed in which all the cellulose in the AIN-93G feed was substituted with γ-CD and demonstrated that, even when WKAH/HkmSlc rats consumed γ-CD, there were no adverse effects such as increases in the levels of AST and ALT and the levels of plasma TG and T-CHO observed in the present study [23,26]. In addition, when male and female Wistar rats were administered a diet containing γ-CD at concentrations ranging from 5% to 20% for 13 weeks, no significant alterations were observed in the levels of AST and ALT or the levels of plasma TG and T-CHO. The safety of γ-CD was subsequently demonstrated based on this report [20,24,25]. However, when γ-CD was ingested during the elevation of 12 OH bile acids in the gastrointestinal tract, the increase in AST and ALT levels and the levels of plasma T-CHO and TG caused by the elevation of 12OH bile acids in the gastrointestinal tract was exacerbated. Consequently, the consumption of γ-CD in an environment where 12OH bile acids are elevated in the gastrointestinal tract, such as during the ingestion of a high-fat diet, may result in the exacerbation of liver injury and dyslipidemia.

Numerous pharmaceutical studies have attempted to improve the bioavailability of hydrophobic drugs by forming inclusion complexes between drugs and CDs [27,28,29]. However, the presence of high concentrations of CDs may reduce the bioavailability of guest molecules with a high affinity for CDs [30]. Thus, the effect of CD on the in vivo absorption of drugs can be either stimulatory or inhibitory, contingent upon the host-guest combination and the molar ratio balance between the two. Furthermore, a study documented the formation of inclusion complexes between CD and primary bile acids, such as CA, or secondary bile acids, such as DCA, which are intestinal bacterial metabolites of primary bile acid (CA) [31]. The elevation of liver injury parameters and the increase in plasma levels of T-CHO and TG observed as a result of the CA+CD diet in this study may be attributed to the formation of stable inclusion complexes between the conjugates of CA and γ-CD in the gastrointestinal tract. This complex formation may inhibit the enterohepatic circulation of bile acids. It is hypothesized that the conjugate of CA and γ-CD forms a stable inclusion complex in the gastrointestinal tract, reaches the cecum, and is subsequently degraded by intestinal microbiota. The CA released from γ-CD may then be metabolized by the intestinal microbiota to produce DCA with high cytotoxicity. There have been reports in the past that increased DCA production is due to a decline in intestinal circulation; for instance, as the efficiency of enterohepatic circulation diminishes with advancing age, CA, which remains unabsorbed in the terminal ileum, is converted to DCA in the large intestine. It has been postulated that this process may contribute to the observed elevation in serum DCA levels [32]. Therefore, we posit that our hypothesis demonstrates sufficient validity, owing to the observed existence of such a phenomenon. Furthermore, the increase in AST and ALT levels in WKAH/HkmSlc rats fed CA for 13 weeks is considered to be related to the increase in the levels of DCA metabolized from CA in the cecum [19]. In addition, when DCA was ingested by WKAH/HkmSlc rats, plasma TG and cholesterol levels increased [19]. Based on these findings, the ingestion of γ-CD when the levels of 12OH bile acids, such as CA, are elevated in the gastrointestinal tract may enhance the elevation in the levels of AST and ALT and plasma T-CHO and TG by stimulating the increased production of DCA in the cecum. That is, the formation of an inclusion complex between γ-CD and CA in the gastrointestinal tract impedes the intestinal circulation of CA. Consequently, an increased quantity of CA reaches the cecum with the inclusion of γ-CD. Upon degradation of γ-CD by the intestinal microbiota in the cecum, it releases a higher amount of CA than usual, which is subsequently metabolized into DCA. This process may enhance an increase in AST and ALT levels and an increase in plasma T-CHO and TG levels. However, to substantiate our hypothesis, it is imperative to perform a comprehensive analysis of bile acid species in both portal blood and cecal contents. This investigation is essential to determine whether the postulated formation of an inclusion complex between γ-CD and CA significantly impedes the intestinal circulation of CA, subsequently leading to the increased production of DCA in the cecum.

Because plasma eicosapentaenoic acid levels increased in the CA+LP and CA+IC groups in the present study, it was assumed that there was no problem with the conversion of α-linolenic acid to eicosapentaenoic acid in the liver. Eicosapentaenoic acid has been reported to reduce elevated T-CHO and TG levels by binding to peroxisome proliferator-activated receptor α (PPARα) [33,34,35]. Based on these findings, elevated eicosapentaenoic acid production resulting from increased α-linolenic acid in the liver, associated with perilla oil consumption, was expected to ameliorate both elevated plasma T-CHO and TG levels induced by the concurrent ingestion of CA or CA+CD diets through the activation of PPARα. The present study showed no significant alteration in plasma T-CHO levels, whereas plasma TG levels exhibited a tendency toward reduction in the CA+LP and CA+IC groups compared with the CA group. In addition, the CA+IC group exhibited a tendency toward reduced plasma T-CHO and TG levels compared with the CA+CD group, with the effect on plasma TG levels being more pronounced than on plasma T-CHO levels. The reason why no statistically significant difference was obtained is thought to be because, in rats fed a diet containing 0.5 g/kg CA, the expression of PPARα decreased and its function declined due to an increase in taurine-CA and taurine-DCA, which are conjugated bile acids derived from CA and DCA, in the liver [36]. In addition, since the transcriptional network initiated by PPARα plays a central role in fatty acid oxidation, its effect on plasma TG levels is thought to be greater than that on plasma T-CHO levels. Therefore, we consider that plasma TG levels exhibit a greater tendency to decrease than plasma T-CHO levels in the CA+LP and CA+IC groups, relative to the CA or CA+CD groups. Based on the aforementioned findings, in the presence of a metabolic abnormality in 12OH bile acid, the resultant metabolic abnormalities in T-CHO and TG levels may not be ameliorated unless a substantial quantity or a potent PPARα ligand is ingested.

The ratio of oleic acid was comparable in the CTRL and CA+LP groups and significantly increased in the CA+IC group compared with in the CTRL and CA+LP groups. A plausible hypothesis for this observation is that the CA+LP group promoted the metabolism of oleic acid to mead acid. However, the enzymatic pathway involved in the metabolism of oleic acid to mead acid is identical to that used in the conversion of α-linolenic acid to eicosapentaenoic acid and linoleic acid to arachidonic acid [37]. Therefore, if the metabolism of oleic acid to eicosapentaenoic acid is enhanced, a comprehensive analysis of the interrelationships among these three pathways is warranted. In addition, the mechanisms by which mead acid influences liver injury and dyslipidemia, along with its overall physiological role, need to be further elucidated.

The ratio of arachidonic acid was significantly reduced in both the CA+LP and CA+IC groups compared with the CTRL group. Relative to the CA group, there was a decreasing trend in the CA+LP group and a significant reduction in the CA+IC group. Conversely, the ratios of α-linolenic acid and eicosapentaenoic acid were significantly higher in the CA+LP and CA+IC groups than in the CTRL and CA+CD groups. Compared with the CA+LP group, the ratio of α-linolenic acid tended to be lower in the CA+IC group and significantly lower in the eicosapentaenoic acid ratio. These findings suggest that the conversion of α-linolenic acid to eicosapentaenoic acid and eicosapentaenoic acid to its metabolites may be enhanced in the CA+IC group compared with in the CA+LP group. Furthermore, this phenomenon may have contributed to the lower ratio of arachidonic acid observed in the CA+IC group than in the CA+LP group. Given that the enzyme groups involved in these two metabolic pathways, α-linolenic acid to eicosapentaenoic acid and linoleic acid to arachidonic acid, are identical [37], it is plausible that the metabolism of linoleic acid was suppressed in the CA+IC group due to the enhanced metabolism of α-linolenic acid in the CA+IC group compared with in the CA+LP group.

Based on the results of the present study, we speculate that resolvins are produced as metabolites of eicosapentaenoic acid [38,39]. This is because resolvin E1, a resolvin derived from eicosapentaenoic acid, tends to reduce obesity-induced increases in serum TG levels and significantly reduces ALT levels [40]. These results are similar to those obtained in the present study. That is, while the increase in ALT levels in the CA group tended to decrease in the CA+LP group, the substantial elevation in ALT levels observed in the CA+CD group was significantly attenuated in the CA+IC group. This differential response is hypothesized to be attributable to the enhanced metabolism of eicosapentaenoic acid to resolvins in the CA+IC group compared with in the CA+LP group. Therefore, future research should investigate whether the enhanced metabolism of eicosapentaenoic acid to resolvins in the CA+IC group effectively ameliorated the marked exacerbation of liver injury observed in the CA+CD group.

Stearoyl-CoA desaturase 1 (SCD1)-mediated palmitate-to-palmitoleic acid metabolism was enhanced in all subjects consuming a diet containing CA. Conversely, the elongation of long-chain fatty acid family member 6 (Elovl6)-mediated metabolism of palmitate to stearate was diminished in all subjects who consumed a diet containing CA. Given that consumption of the CA diet elicits an increase in sterol regulatory element-binding protein-1c (SREBP-1c) expression [19], the expression of SCD1 and Elovl6, the downstream targets of SREBP-1c, is expected to be upregulated [41,42]. However, the results obtained in the present study suggest that SCD1 may exhibit a higher level of upregulated expression or enhanced enzymatic activity with a CA diet intake than with Elovl6. Furthermore, the present study showed that the CA+CD group, with the highest AST levels, had the highest plasma palmitoleic acid levels, whereas the CTRL group, with the lowest AST levels, had the lowest plasma palmitoleic acid levels. A positive association has been reported between plasma palmitoleic acid levels and AST levels in patients with non-alcoholic fatty liver disease (NAFLD) [43]. Thus, γ-CD ingestion in the presence of elevated levels of 12OH bile acids in the gastrointestinal tract may enhance the conversion of palmitic acid to palmitoleic acid, thereby exacerbating liver injury. This mechanism potentially involves the enhanced activation of the SREBP-1c/SCD1 pathway associated with increased hepatic SREBP-1c expression. That is, the hepatic expression of SREBP-1c may be elevated in the CA+CD group compared with in the CA group.

## 4. Materials and Methods

### 4.1. Reagents

γ-CD was purchased from CycloChem Co., Ltd. (Tokyo, Japan), and cold-pressed perilla oil was purchased from the Okuizumo Nakamura Farm Co., Ltd. (Okuizumo, Japan). Free and esterified FAs in rat plasma were converted to methyl esters using reagent kits following the manufacturer’s protocol (Nacalai Tesque Inc., Kyoto, Japan). All other chemicals were reagent grade (Fujifilm Wako Pure Chemical Corporation, Osaka, Japan). Inclusion complexes of perilla oil and γ-CD were prepared according to a previously described method [23,26].

### 4.2. Animals

All animal experiments and procedures were approved by the Animal Care and Use Committee of Shimane University (approval number: MA28-3). With regard to the housing, husbandry, and handling of mice, we adhered to the Institutional Regulations of Shimane University, established in accordance with the Act on Welfare and Management of Animals (Act No. 105) and relevant standards and guidelines in Japan. Male Wistar King A Hokkaido rats (WKAH/HkmSlc; Japan SLS, Kyoto, Japan) were housed in individual plastic cages (total per group, *n* = 8) in a temperature-controlled room at 26 ± 1 °C, 55 ± 5% humidity, and a 12 h light/dark cycle. All the rats had free access to water and food throughout the study period. Body weight and food intake were assessed every alternate day. Following the treatment period, the abdominal aorta blood of the rats was collected using a syringe containing heparin (final concentration of 50 U/mL blood) and aprotinin (final concentration of 500 kIU/mL blood) under anesthesia with 5% isoflurane for induction and 2% for maintenance via a nose cone. In addition, the liver and epididymal fat were collected and weighed. Plasma was prepared by centrifuging the collected abdominal aorta blood at 2000× *g* for 10 min at 4 °C. Each prepared plasma sample was stored at −80 °C until analysis.

### 4.3. Blood Plasma Analysis

AST (IU/L), ALT (IU/L), T-CHO (mg/dL), TG (mg/dL), and GLU (mg/dL) levels were measured using commercially available kits on a Hitachi 7180 Autoanalyzer (Hitachi Ltd., Tokyo, Japan) at the Nagahama Institute for Biochemical Science, Oriental Yeast Co., Ltd. (Shiga, Japan).

### 4.4. Plasma Fatty Acid Analysis by GC-MS

Plasma fatty acids were analyzed using a Shimadzu GCMS-QP2010 Ultra spectrometer equipped with a quadrupole mass detector (1.00 ± 0.10 kV) and a DB-5ms 30 m × 0.25 mm i.d. capillary column with a film thickness of 0.25 µm (Agilent Technologies, Santa Clara, CA, USA), as previously described [26].

### 4.5. Statistical Analysis

Differences between groups in vivo were compared using one-way ANOVA with Tukey–Kramer post hoc analysis. To verify the results of this study with those of a previous study [19], the Student’s t-test was used to compare the CTRL and CA groups. The mean difference was considered statistically significant at *p* < 0.05. The results were reported as mean ± standard error (SE) because SE allows for interval estimation of the population mean.

## 5. Conclusions

The findings of the present study suggest that the safety of γ-CD requires verification using both healthy laboratory animals and specifically designed pathological models. Consequently, future studies should focus on assessing the safety of γ-CD in various pathological models. In addition, a similar verification of the safety of γ-CD in humans is necessary. The results also indicate that compounds with γ-CD-binding properties may potentially mitigate the adverse effects associated with γ-CD ingestion. Given the widespread use of γ-CD in food and pharmaceutical products, careful consideration of the compounds present in γ-CD is extremely important.

## Figures and Tables

**Table 1:** Food formulations for the experimental diets.

Composition (Weight %)	CTRL	CA	CA+LP	CA+CD	CA+IC
Corn Starch	39.75	39.75	39.75	39.75	39.75
Casein	20.00	20.00	20.00	20.00	20.00
Maltodextrin	13.20	13.20	13.20	13.20	13.20
Sucrose	10.00	9.95	9.95	9.95	9.95
Soybean Oil	7.00	7.00	6.12	7.00	6.12
Perilla Oil	–	–	0.88	–	–
Cellulose	5.00	5.00	5.00	–	–
γ-CD	–	–	–	5.00	–
Inclusion Complex	–	–	–	–	5.88
Mineral Mix	3.50	3.50	3.50	3.50	3.50
Vitamin Mix	1.00	1.00	1.00	1.00	1.00
L-Cystine	0.30	0.30	0.30	0.30	0.30
Choline Bitartrate	0.25	0.25	0.25	0.25	0.25
Cholic Acid	–	0.05	0.05	0.05	0.05

**Table 2:** Plasma fatty acid composition (%) for each treatment.

Fatty Acids	CTRL	CA	CA+LP	CA+CD	CA+IC
Palmitic acid	24.2 ± 0.4	23.2 ± 0.4	23.2 ± 0.3	23.3 ± 0.4	24.3 ± 0.2
Palmitoleic acid	2.1 ± 0.4 ^b^	4.4 ± 0.3 ^a^	4.5 ± 0.2 ^a^	5.5 ± 0.4 ^a^	4.5 ± 0.3 ^a^
Stearic acid	9.7 ± 0.3 ^a^	7.6 ± 0.2 ^b^	7.7 ± 0.2 ^b^	7.1 ± 0.2 ^b^	7.4 ± 0.2 ^b^
Oleic acid	14.4 ± 0.5 ^c^	16.2 ± 0.3 ^ab^	14.8 ± 0.3 ^bc^	16.3 ± 0.3 ^ab^	16.3 ± 0.4 ^a^
Linoleic acid	23.4 ± 1.0	25.0 ± 0.9	25.0 ± 0.8	24.3 ± 0.9	25.2 ± 0.6
α-Linolenic acid	1.1 ± 0.1 ^b^	1.4 ± 0.1 ^b^	3.4 ± 0.3 ^a^	1.6 ± 0.1 ^b^	2.9 ± 0.2 ^a^
Arachidonic acid	20.5 ± 1.2 ^a^	17.4 ± 1.0 ^ab^	15.2 ± 0.8 ^bc^	16.5 ± 0.9 ^bc^	13.5 ± 0.8 ^c^
Eicosapentaenoic acid	0.8 ± 0.1 ^c^	0.7 ± 0.1 ^c^	2.2 ± 0.2 ^a^	0.9 ± 0.1 ^c^	1.7 ± 0.1 ^b^
Docosahexaenoic acid	2.2 ± 0.2	1.7 ± 0.2	2.0 ± 0.1	2.1 ± 0.2	2.0 ± 0.0

Data are shown as the mean ± SE. Means within a row with different superscript letters are significantly different from each other at *p* < 0.05.

## Data Availability

The data presented in this study are available on request from the corresponding author.
